# LncRNA PITPNA-AS1 as a Potential Diagnostic Marker and Therapeutic Target Promotes Hepatocellular Carcinoma Progression via Modulating miR-448/ROCK1 Axis

**DOI:** 10.3389/fmed.2021.668787

**Published:** 2021-05-12

**Authors:** Qing-fang Wang, Qing-lin Wang, Ming-bo Cao

**Affiliations:** ^1^Department of Clinical Laboratory, The First Affiliated Hospital of Zhengzhou University, Zhengzhou, China; ^2^Department of Pharmacology, The First Affiliated Hospital of Zhengzhou University, Zhengzhou, China; ^3^Department of Telemedicine Center, The First Affiliated Hospital of Zhengzhou University, Zhengzhou, China

**Keywords:** LncRNA PITPNA-AS1, miR-448, ROCK1, hepatocellular carcinoma, biomarker

## Abstract

**Background:** Long non-coding RNAs are critical to hepatocellular carcinoma (HCC) developments. LncRNA PITPNA antisense RNA 1 (PITPNA-AS1) is a new regulator in several tumors. However, the mechanism by which PITPNA-AS1 mediates the tumorigenesis of HCC remains unclear.

**Methods:** RT-qPCR was used to detect the level of PITPNA-AS1 in HCC specimens and cells. The biological functions of PITPNA-AS1 were explored by several functional experiments *in vivo* and *in vitro*. The binding relationship among PITPNA-AS1, miR-448 and ROCK1 were studied by Luciferase assay and pull-down assays.

**Results:** We found that PITPNA-AS1 expressions were distinctly upregulated in both HCC specimens and cell lines. High PITPNA-AS1 levels were an unfavorable biomarker for patients with HCC. Functionally, knockdown of PITPNA-AS1 suppressed the proliferation, migration and invasion of HCC cells. Mechanistically, PITPNA-AS1 functioned as competing endogenous RNA to increase ROCK1 expressions via sponging miR-448.

**Conclusion:** The newly identified PITPNA-AS/miR-448/ROCK1 axis promoted the oncogenicity of HCC cells. This novel axis is likely to be a promising HCC therapeutic aim.

## Introduction

Hepatocellular carcinoma (HCC) is a common malignancy worldwide and the second leading cause of cancer-related death (1). Several researches have indicated that HCC morbidity is primarily caused by environmental pollution, alcohol addiction, liver cirrhosis and hepatitis B/C virus infecting process (2). Even though effective surgical technique and diagnostic processes have resulted in the distinct improvements of clinical outcome of HCC patients, the long-term survivals are still unsatisfactory largely due to positive metastasis and the high recurrence (45–65% at 5 years) (3, 4). Thus, the molecular systems within HCC progression should be explored in depth for the improvements of diagnosis and clinical treatments of HCC.

Long non-coding RNAs (lncRNAs) are non-protein coding transcripts longer than 200 nucleotides (5). Many lncRNAs modulate gene expressions at several levels including transcription and posttranscriptional processing (6). More and more evidences have demonstrated the involvements of lncRNAs in a variety of disease states. According to a growing volume of literature, the dysregulation of lncRNAs was frequent in various tumors and involved in tumor progression (7, 8). Functional lncRNAs have been identified to serve as tumor promotors or anti-oncogenes, which suggests their potential used as novel tumor biomarkers and potential therapeutic targets (9). However, the expression and underlying mechanism of HCC associated with aberrant lncRNAs remain largely unclear.

When we analyzed TCGA datasets, we found an overexpressed lncRNA, PITPNA-AS1, within HCC. PITPNA-AS1, located on 17p13.3, exhibited a high level in several different types of tumors. In recent years, several researches reported the expression and tumor-related function of PITPNA-AS1 in several tumors, including HCC, lung tumor and colorectal tumor (10–12). However, the roles and related mechanisms of PITPNA-AS1 in the initiation and progressions of HCC were rarely reported.

## Materials and Methods

### Cases and Samples

Ninety-three sets of HCC tissues and adjacent normal hepatic tissues were obtained from patients with HCC who underwent HCC resection at the First Affiliated Hospital of Zhengzhou University between July 2015 and November 2017. No chemotherapy and radiotherapy were performed before the surgery. By complying with the 2009 American Joint Committee for Cancer staging system, histological and pathological diagnostics for patients with HCC were determined. The present work received the approving process from Institute Research Medical Ethics Committee of the First Affiliated Hospital of Zhengzhou University. The respective case presented the consent in an informed manner prior to the research.

### Cell Lines and Transfection

Liver normal cell lines L02 and HCC cell lines (including Hep3B, HepG2, SMMC-7721 as well as HCCLM3) of humans were provided by Shanghai Institute of Cell Biology (Shanghai, China). RPMI 1640 medium involving 10 % fetal bovine serum (Bieyu Technology, Nanjing, Jiangsu, China), 100 U/ml penicillin, and 100 mg/ml streptomycin was used to culture cells in humidified air with 5 % CO_2_ at the temperature of 37°C.

Short hairpin RNA (shRNA) sequences aiming PITPNA-AS1 were designed by OriGene Company (Hiadian, Beijing, China). The shRNAs were inserted into lentiviral pHBLV/U6-Scramble-Luc-Puro01 vector (Biomart Technology, Haidian, Beijing, China), termed as sh-PITPNA-AS1-1 as well as sh-PITPNA-AS1-2; negative control was named sh-NC. MiR-448 mimics and miR-448 inhibitors together with their controls were provided by Ziqibo Technology (Xuhui, Shanghai, China). The present work adopted Lipofectamine 2000 (Invitrogen, Chengdu, Sichuan, China) for performing plasmid transfections based on the producer's protocol. Western blot assays or RT-qPCR was used to examine transfection efficiency.

### Bioinformatics Analysis

“GEPIA” (http://gepia.cancer-pku.cn/) analyzed the expressing state and clinical significance pertaining to PITPNA-AS1 in HCC. The binding sites among PITPNA-AS1, miR-448 and ROCK1 received the prediction on starBase (http://starbase.sysu.edu.cn/index.php).

### Quantitative Real-Time PCR Assay

Total RNA was extracted from tissues or cultured cells with TRIzol (Invitrogen). cDNA synthesis was performed with 2 μg of overall RNA, using the miScript II RT Tool (Qiagen) by complying with the directions of the producer. The expressing states pertaining to lncRNA and genes received the assessment based on one ABI7500 Real-Time PCR Process (Applied Biosystems) as well as the SYBR-Green PCR Master Mix Tool (Takala, Hangzhou, Zhejiang, China). The results received the normalization to expressing states pertaining to U6 or GAPDH. Comparative quantification was determined using the 2^−ΔΔCt^ methods. All primer sequences are summarized in Table 1.

**Table 1:** Primers for qPCR assays.

**Names**	**Sequence (5^**′**^-3^**′**^)**
PITPNA-AS1: forward	GACCACACATTCACCCTCAT
PITPNA-AS1: reverse	CTTACTCACCGTTGCCACCCAC
miR-448: forward	TCGGCAGGTTGCATATGTAGGA
miR-448: reverse	CTCAACTGGTGTCGTGGA
ROCK1: forward	AACATGCTGCTGGATAAATCTGG
ROCK1: reverse	TGTATCACATCGTACCATGCCT
U6: forward	CTCGCTTCGGCAGCACA
U6: reverse	AACGCTTCACGAATTTGCGT
GAPDH: forward	GGAGCGAGATCCCTCCAAAAT
GAPDH: reverse	GGCTGTTGTCATACTTCTCATGG

### Cell Counting Kit-8 (CCK-8) Assay

Cells proliferation was examined by the use of the cell proliferation reagent WST-8 (Absin, Pudong, Shanghai, China). Within 24-well plates based on 3 × 10^5^ cells/well density, cells received the plating process. Next, within 10% CCK-8 under the dilution by using common culture medium, HCC cells received the incubation at 37°C to achieve color conversion. Based on Elx800 Reader, Absorbance (A) then received the recording process at 450 nm.

### Colony Formation Assay

At a density of 100 cells/well within 6-well plates, HCC cells received the seeding process. When cells received the 2-weeks incubating process, they underwent the PBS-based cleaning process two times, followed by the methanol-based fixing process and the staining process based on crystal violet. Based on a microscope, the present study made a counting of the colonies covering >50 cells.

### Wound Healing Assays

Indicated HCC cells were plated in 6-well plates and cultured at 37°C. Once cells were attached completely, they were scraped to form a wound in the middle of the plates. Washed with PBS three times, DMSO and 3c compound was added in duplicate. After final incubation, pictures were again taken under microscope.

### Transwell Assays

Cells (5 × 10^4^) received the suspending process within serum-free DMEM and then the addition into chambers (8 mm, BD Biosciences) under the coating of BD BioCoat Matrigel. When the incubating process was achieved, by employing one cotton tip, the cells onto the surface of the upper membrane received the removal. The RPMI 1640 medium involving 10% FBS received the addition into the bottom chamber. Cells under the migrating or invading process within the bottom chamber received the 0.1% crystal violet-based staining process. Cells were then counted under an optical microscope.

### EdU Staining

Cells (5 × 10^4^) were suspended in serum-free DMEM and added to chambers (8 mm, BD Biosciences) coated with BD BioCoat Matrigel. Subsequently, 5 × 10^4^ cells underwent the 4% paraformaldehyde-based fixing process, 0.5% Troxin X-100 based incubating process, as well as 1 × Apollo® 488 fluorescent staining. For determining EdU-positive cells' percentage within 5 random fields in the respective well, this study employed fluorescent microscopy (Nikon, Japan).

### Subcellular Fractionation

By employing a PARIS tool (AM1921, Invitrogen, Wosheng Biology, Hangzhou, Zhejiang, China) by complying with the guidelines of the producers, this study conducted the nuclear and cytosolic part separating process.

### RNA Pull-Down Assays

After cells were quantitated, our group employed 1 ml of cell lysis buffer for 72 h treating of cells. Next, cells received the rotating process overnight at 4°C after adding 500 pM antisense oligos and 2 μl of RNase inhibitors (Keming Technology, Haidian, Beijing, China). Cell lysis buffer was applied to wash beads five times. Total RNAs were subjected to RT-PCR assays.

### Luciferase Reporter Assays

pMiR-Reporter Vector and DNA oligonucleotide received the utilization for constructing the report vector of PITPNA-AS1 wild type/mutant (PITPNA-AS1-WT/MUT) and ROCK1 wild type/mutant (ROCK1-WT/MUT). Subsequently, the above two vectors received the co-transfecting process by using miR-448 mimics and negative control mimics (NC mimics), respectively, and then co-transfected into HCCLM3 and HepG2 cells. Forty-eight hours after transfection, the luciferase activity was detected under a dual-luciferase reporter assay system (Promega).

### Xenograft Tumor Model

BALB/c female nude mice according to Vital River Company (Chaoyang, Beijing, China) were used for *in vivo* experiments. HepG2 cells under the stable transfection by employing sh-PITPNA-AS1-1 or sh-NC received the subcutaneous injection in nude mice's right flank (*n* = 6). Samples received the euthanizing process, and the xenografts underwent the dissecting and weighing process based on 28-days injecting process. Tumor volume received the calculation by (length × width^2^)/2. The animal-related protocol was approved by the Animal Research Ethics Committee of the First Affiliated Hospital of Zhengzhou University.

### Western Blot

Overall protein received the extraction based on the Total Protein Extraction Tool (Abnova, Aimeijie, Wuhan, Hubei, China). Based on the BCA approach, the contents pertaining to the protein samples received the determination, and 40 μg protein according to the respective sample underwent the treatment with 10% SDS-PAGE. Then the proteins received the transfer to Millipore 0.45 μM polyvinylidene difluoride (PVDF) membrane. When the samples received the incubating process by employing antibodies specific in terms of E-cadherin, N-cadherin, Vimentin, ROCK1 and GAPDH, the blots received the incubating process by employing anti-mouse or goat anti-rabbit secondary antibodies. All antibodies were purchased from Qiangyao Technology (Pudong, Shanghai, China). Signals were visualized using ECL Substrates (Pierce). GAPDH served as an endogenous control.

### Statistical Analysis

The present study employed SPSS v. 20.0 software package (IBM Corp., Armonk, NY, USA) to statistically investigate the information acquired. The group differences were compared using the ANOVA test or student's *t*-test. The total surviving states pertaining to HCC cases received the assessment based on the Kaplan-Meier curve, and the distinction within total surviving states received the determination with the use of log-rank methods. Multivariate Cox regression was performed on each clinical covariate to examine its influence on patient survival. P < 0.05 was regarded as statistically significant.

## Results

### Increased PITPNA-AS1 Expression in HCC Tissues

**Figure 1:**
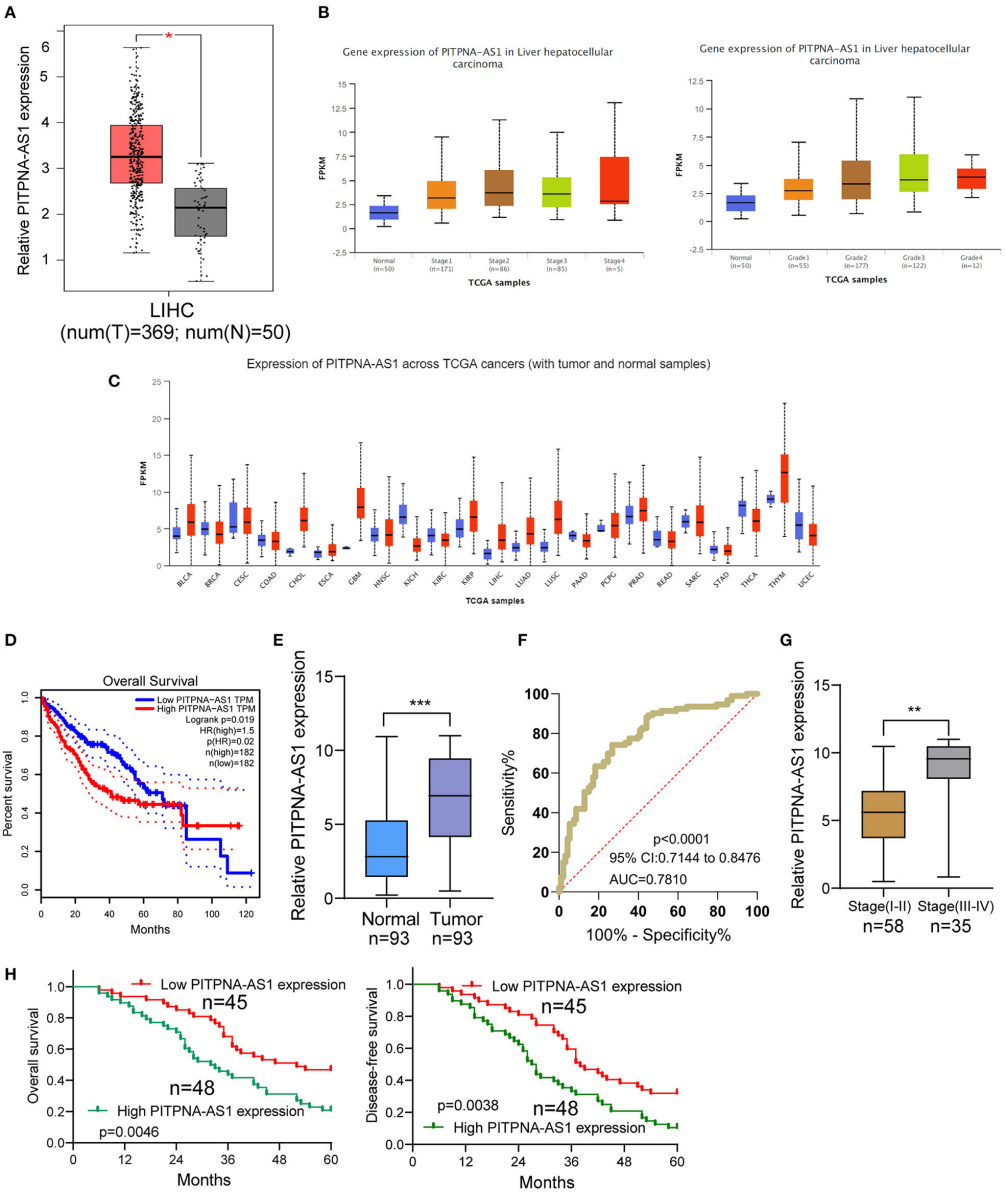
PITPNA-AS1 is upregulated in HCC tissues and is associated with poor prognosis. **(A)** The distinct upregulation of PITPNA-AS1 expression in HCC specimens (*n* = 369) compared to non-tumor specimens (*n* = 50) from TCGA datasets. **(B)** The expressing pattern of PITPNA-AS1 in HCC specimens with different stages. **(C)** Based on TCGA datasets, Pan-cancer expressions of PITPNA-AS1 were shown. The red column represented the tumor samples and the blue column represented the normal samples. **(D)** Kaplan-Meier curves for survival time in HCC patients divided according to PITPNA-AS1 expression based on TCGA datasets. **(E)** RT-PCR for the determination of PITPNA-AS1 expression in 93 pairs of HCC specimens and non-tumor tissues. **(F)** ROC curves analysis for the HCC diagnostic capability of PITPNA-AS1 expression. **(G)** Higher levels of PITPNA-AS1 were observed in HCC specimens with advanced stages than those with early stages. **(H)** Kaplan-Meier curves for overall survival and disease-free survival in patients with HCC divided according to PITPNA-AS1 expression. ***P < 0.001, **P < 0.01.

For screening functional lncRNAs in HCC progression, we analyzed TCGA datasets. We found a lncRNA, PITPNA-AS1 which was distinctly overexpressed in HCC specimens from TCGA datasets (Figure 1A). Advanced HCC specimens also showed higher levels of PITPNA-AS1 than those with early stages (Figure 1B). A pan-cancer analysis indicated the overexpressed trend of PITPNA-AS1 in many different types of tumors (Figure 1C). Based TCGA datasets, patients with high PITPNA-AS1 showed a shorter overall survival than those with low PITPNA-AS1 expression (Figure 1D). These results highlighted the involvements of PITPNA-AS1 in HCC progression. Then, we performed RT-PCR in our cohort and observed that PITPNA-AS1 expression in HCC specimens was distinctly higher than that in the matched non-tumor specimens (Figure 1E). ROC assays confirmed the diagnostic value of high PITPNA-AS1 expression in distinguishing HCC specimens from normal tissues with an AUC = 0.7810 and *p* < 0.001 (Figure 1F). Higher levels of PITPNA-AS1 were observed in HCC specimens with stage (III-IV) than those with stage (I-II) (Figure 1G). Overall, our findings suggested PITPNA-AS1 as a possible functional lncRNA in HCC progression.

### Prognostic Values of PITPNA-AS1 Expression in HCC Patients

Using the mean value of PITPNA-AS1 expression level (6.87) as the cut-off value, 93 samples were divided into two groups (high expression and low expression). We observed that high PITPNA-AS1 expression was associated with larger tumor size (*p* = 0.004), tumor differentiation (*p* = 0.021), positive lymph node metastasis (*p* = 0.012), and advanced clinical stage (*p* = 0.011) (Table 2). To explore whether the dysregulation of PITPNA-AS1 may influence the survivals of HCC patients, we performed Kaplan-Meier methods, finding that the 5-years overall survival (*p* = 0.0046) and disease-free survival (*p* = 0.0038) of high PITPNA-AS1 expression group were distinctly shorter than those of low PITPNA-AS1 expression group (Figure 1H). More importantly, Multivariate analysis revealed that high PITPNA-AS1 expression was an independent prognostic factor for both OS (HR = 3.241, 95% CI: 1.376–5.467, *P* = 0.005) and DFS (HR = 3.413, 95% CI: 1.457–5.774, *P* = 0.002, Table 3).

**Table 2:** Relationship between PITPNA-AS1 expression and clinicopathological characteristics.

**Parameters**	**Group**	**Total**	**PITPNA-AS1**		***p*-value**
			**expression**		
			**High**	**Low**	
Gender	Male	53	28	25	0.787
	Female	40	20	20	
Age (years)	<50	44	23	21	0.904
	≥50	49	25	24	
Tumor size (cm)	<4	52	20	32	0.004
	≥4	41	28	13	
Tumor differentiation	Well/Moderate	57	24	33	0.021
	Poor	36	24	12	
Lymph node metastasis	Absence	65	28	37	0.012
	Presence	28	20	8	
Clinical stage	I-II	58	24	34	0.011
	III-IV	35	24	11	

**Table 3:** Multivariate analyses of prognostic factors in HCC patients.

**Variables**	**Overall survival**			**Disease-free survival**		
	**HR**	**95% CI**	***p*-value**	**HR**	**95% CI**	***p*-value**
Gender	0.783	0.456–1.783	0.342	0.974	0.545–2.013	0.251
Age (years)	0.978	0.554–2.132	0.432	1.241	0.678–2.342	0.268
Tumor size	1.453	0.832–2.342	0.134	1.554	0.942–2.456	0.117
Tumor differentiation	3.113	1.342–4.443	0.014	3.241	1.432–4.765	0.008
Lymph node metastasis	3.344	1.452–6.773	0.001	3.563	1.677–7.347	0.001
Clinical stage	2.896	1.327–5.138	0.012	3.183	1.442–5.348	0.008
PITPNA–AS1 expression	3.241	1.376–5.467	0.005	3.413	1.457–5.774	0.002

### Silencing of PITPNA-AS1 Suppresses the Proliferating Process and Metastasizing Process Pertaining to HCC Cells

**Figure 2:**
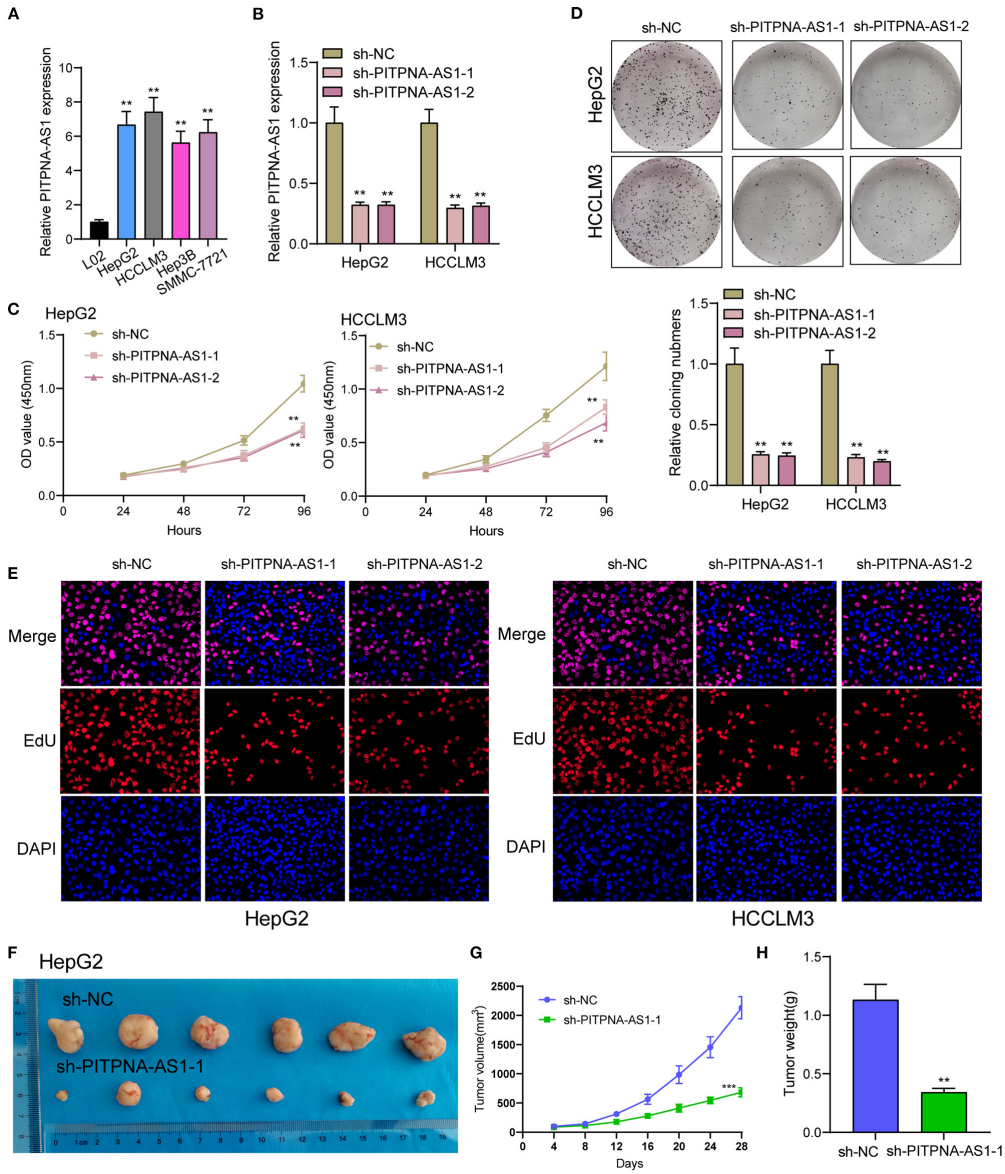
PITPNA-AS1 promotes HCC cell proliferation *in vitro* and *in vivo*. **(A)** The expression in four HCC cells and LO2 cells was determined by RT-PCR. **(B)** RT-PCR confirmed the transfection efficiency of sh-PITPNA-AS1-1 and sh-PITPNA-AS1-2. **(C)** CCK-8 assays were performed to detect the proliferative abilities of the transfected HepG2 and HCCLM3 cells. **(D)** A colony-forming growth assay was performed to determine the proliferation of HepG2 and HCCLM3 cells after treatments. **(E)** EDU staining assays were applied to detect the proliferation of sh-PITPNA-AS1-1 and sh-PITPNA-AS1-2-transfected HCC cells. **(F)** Total number of tumors after removal from the mice. **(G)** Tumor growth curve. HepG2 cells were transfected with sh-NC or sh-PITPNA-AS1-1, and then injected into nude mice (*n* = 6), respectively. **(H)** Tumor weight were represented. ***P < 0.001, **P < 0.01.

**Figure 3:**
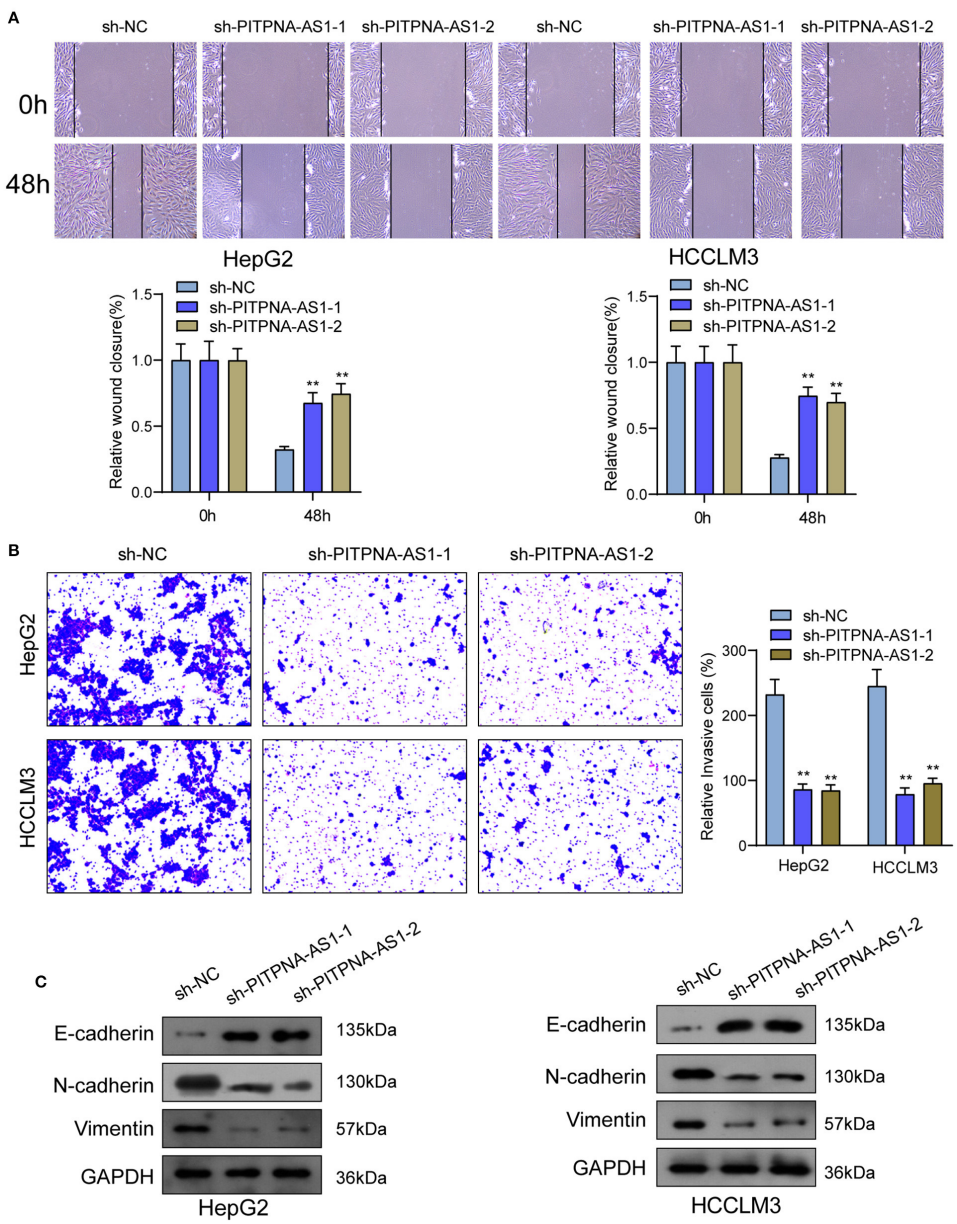
PITPNA-AS1 knockdown inhibited cell migration and invasion of HCC cells. **(A)** The migration abilities of tumor cells were assessed by wound healing assays. **(B)** The number of invasion cell was detected by Transwell assays. **(C)** Western blotting was applied to detect the expression of EMT-related proteins in HepG2 and HCCLM3 cells. **P < 0.01.

We examined the levels of PITPNA-AS1 within HCC cells and found that PITPNA-AS1 expression was upregulated in four HCC cells (Figure 2A). HepG2 and HCCLM3 cells with highest PITPNA-AS1 levels were chosen for further study. This study carried out RT-PCR for detecting the efficient property of the transfecting process pertaining to sh-PITPNA-AS1-1/-2/NC. According to the results, PITPNA-AS1 expression decreased distinctly when sh-PITPNA-AS1-1/-2 was transfected in HepG2 and HCCLM3 cells (Figure 2B). The growth curves obtained from CCK8 proliferation assay indicated that PITPNA-AS1 knockdown significantly inhibited cell proliferation and colony formation in HCC cells (Figures 2C,D). EdU assays were performed and the results showed that PITPNA-AS1 knockdown significantly reduced the number of Edu-positive cells compared with that among the control cells (Figure 2E). The results of *in vivo* assays also confirmed that volume and weight of tumors in the sh-PITPNA-AS1 (PITPNA-AS1 knockdown) group were significantly smaller than the control group (Figures 2F–H). To further explore whether the dysregulation of PITPNA-AS1 exhibited effects on metastasis abilities of HCC cells, Transwell tests and Wound healing tests were carried out. According to the result, knockdown of PITPNA-AS1 limited HCC cells from migrating and invading (Figures 3A,B). The present study employed for Western blotting detecting the expressions pertaining to proteins associated with EMT, and the information proved that the down-regulation of PITPNA-AS1 elevated E-cadherin expression and down-regulated Vimentin and N-cadherin expressions (Figure 3C).

### PITPNA-AS1 Acted as ceRNA of miR-448 in HCC

**Figure 4:**
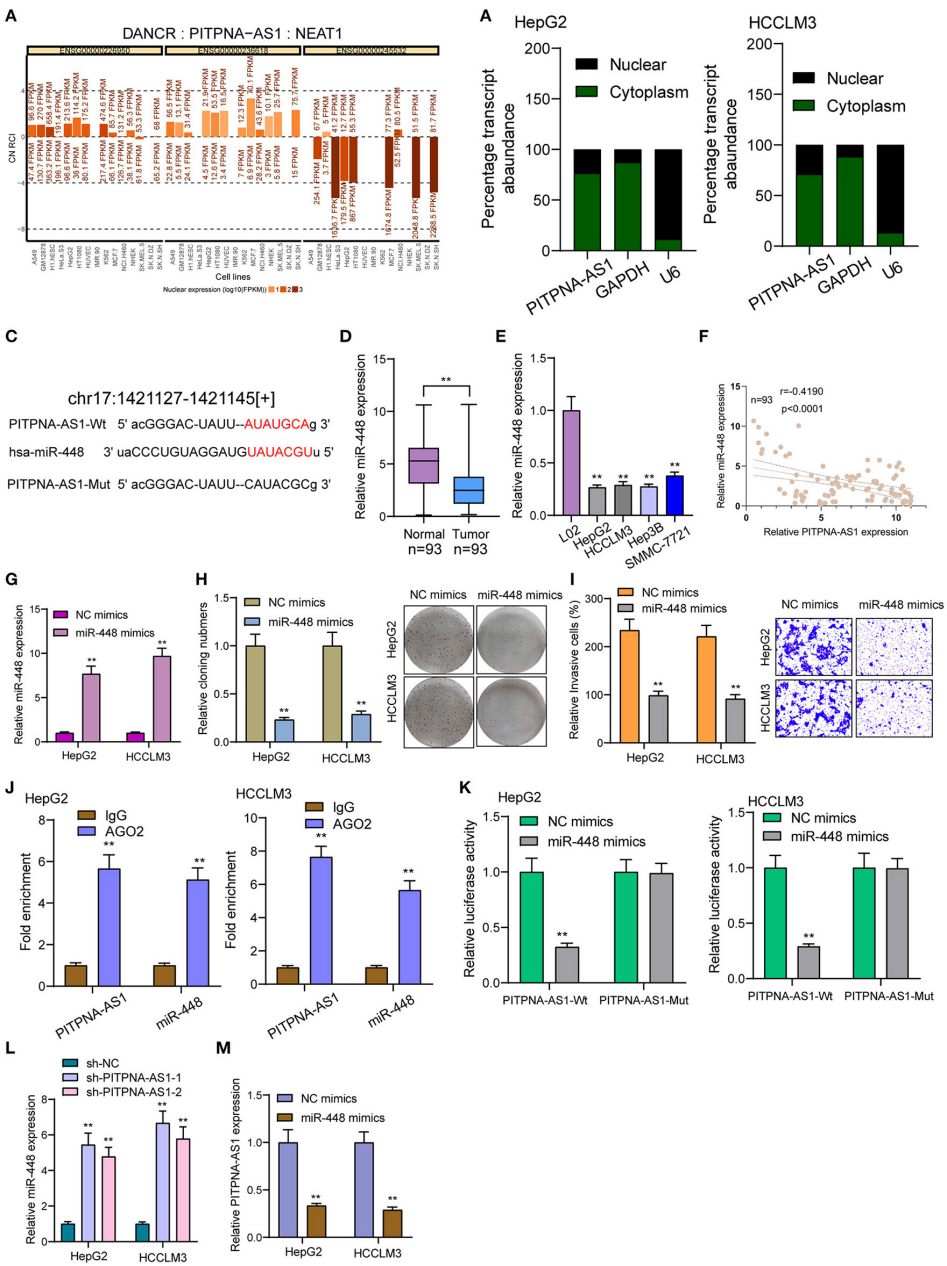
PITPNA-AS1 functioned as a sponge of miR-448. **(A)** “lncATLAS” showed the subcellular localization of PITPNA-AS1 in a series of tumor cells. **(B)** Relative PITPNA-AS1 expression levels in nuclear and cytosolic fractions of HepG2 and HCCLM3 cells. **(C)** Schematic outlining the predicted binding sites between PITPNA-AS1 and miR-448. **(D,E)** RT-PCR determined the expression of miR-448 in HCC specimens and cell lines. **(F)** Correlation analysis between PITPNA-AS1 expression and miR-448 expression in 93 HCC specimens. **(G)** Knockdown of miR-448 in HepG2 and HCCLM3 cells after the transfection of mi-448 mimics. **(H)** Clone formation assay showed the clone number of HCC cells transfected with miR-448 mimics and NC mimics. **(I)** Transwell assays were used to determine the effects of mi-448 overexpression on HepG2 and HCCLM3 cells invasion. **(J)** RNA pull-down assays were performed for the determination of combination between miR-448 and PITPNA-AS1. **(K)** Luciferase reporter assay showed the activity within miR-448 and PITPNA-AS1 wild type or mutant. **(L)** miR-448 expression was deceased in HepG2 and HCCLM3 cells transfected with sh-PITPNA-AS1-1 or sh-PITPNA-AS1-2. **(M)** miR-448 overexpression resulted in the suppression of PITPNA-AS1 expression in HepG2 and HCCLM3 cells. **P < 0.01.

By competitively binding microRNAs, cytoplasmic lncRNAs act as competing endogenous RNAs (ceRNAs) to exhibit their regulator functions. According to the lncATLAS website (http://lncatlas.crg.eu/), PITPNA-AS1 was primarily distributed in the cytoplasm (Figure 4A). Using subcellular fractionation, we also observed that a larger proportion of PITPNA-AS1 was in the cytoplasm (Figure 4B). Based on online prediction algorithm (starBase v2.0), miR-448 was estimated to involve a putative binding site with PITPNA-AS1 (Figure 4C). As revealed from the results of RT-PCR, miR-448 expression was distinctly down-regulated in both HCC specimens and cell lines (Figures 4D,E). Correlation analysis revealed that PITPNA-AS1 expression was negatively associated with miR-448 in 93 HCC tissues (Figure 4F). Then, we explored the function of miR-448 in HCC cells, and miR-448 overexpressing state was found to limit HCCLM3 and HepG2 cells from being proliferated and invaded (Figures 4G–I). RNA-pull down assays suggested the combination between miR-448 and PITPNA-AS1 in HCC cells (Figure 4J). According to Luciferase reporter assays, miR-448 mimics noticeably limited PITPNA-AS1-WT's luciferase activity, whereas it failed to alter PITPNA-AS1-MUT's relative luciferase activity (Figure 4K). Finally, we observed that knockdown of PITPNA-AS1 resulted in the distinct suppression of miR-448, while overexpression of miR-448 exhibited a contrary result (Figures 4L,M).

### PITPNA-AS1 Upregulates ROCK1 Expression by miR-448

**Figure 5:**
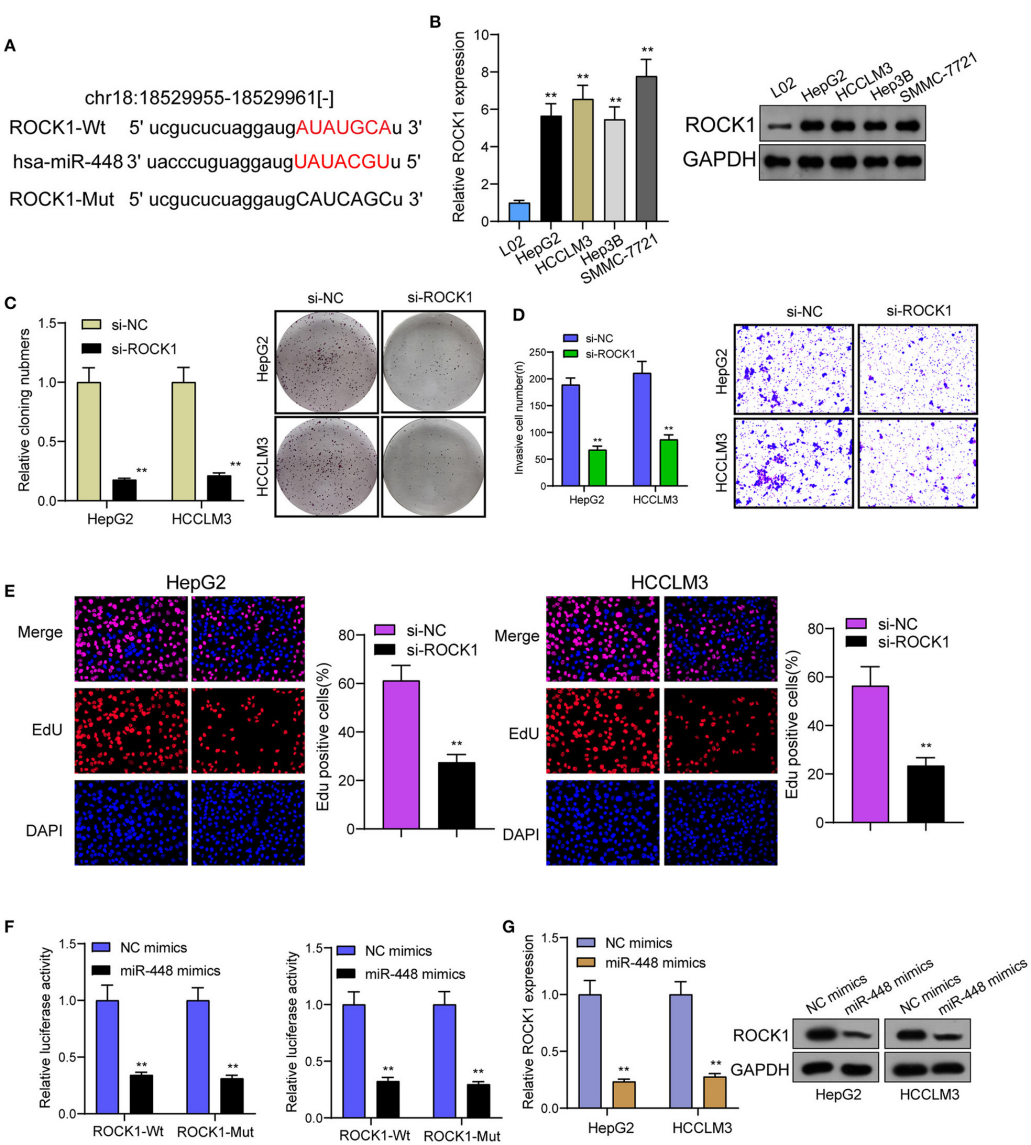
miR-448 regulated ROCK1 expression in HCC cells. **(A)** The predicted miR-448 target site in the 3′UTRof ROCK1 mRNA and its mutated version. **(B)** ROCK1 levels in different HCC cell lines were measured by RT-PCR and western blot. **(C)** The cell proliferation in response to ROCK1 knockdown was examined using colony formation analysis. **(D)** Transwell assays were conducted to measure cell invasion. **(E)** EdU were performed to detect cell proliferation after knockdown of ROCK1. **(F)** miR-448 significantly suppressed the luciferase activity that carried wild-type ROCK1 but not the mutant ROCK1. **(G)** Overexpression of miR-448 suppressed the expression of ROCK1 in HepG2 and HCCLM3 cells. **P < 0.01.

**Figure 6:**
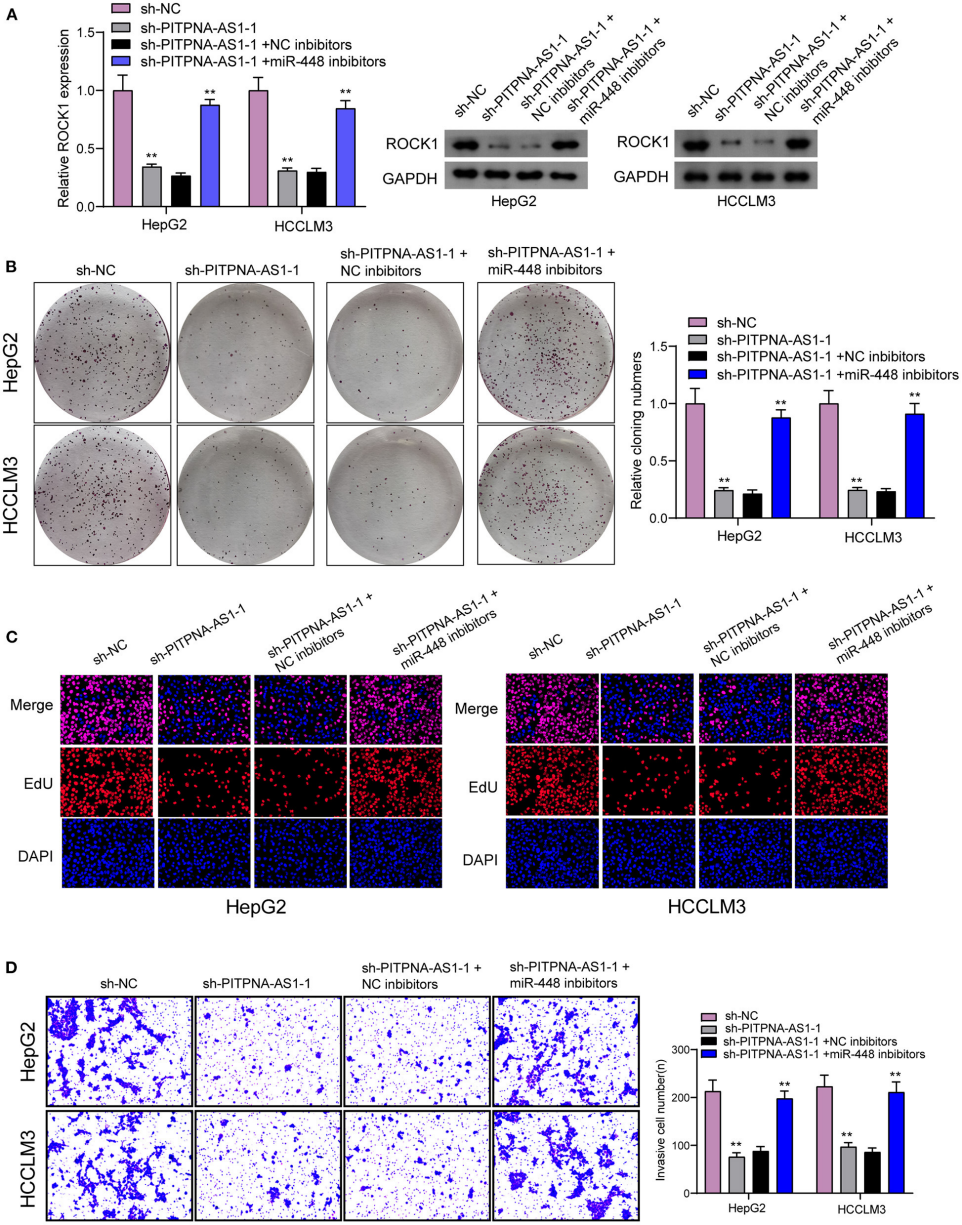
PITPNA-AS1 promoted HCC progression via decreasing ROCK1 through sponging miR-448. **(A)** The expression of ROCK1 in HepG2 and HCCLM3 cells transfected with sh-NC, sh-PITPNA-AS1-1, sh-PITPNA-AS1-1+NC inhibitors or sh-PITPNA-AS1-1+ miR-448 inhibitors. **(B–D)** colony-forming growth assay, Edu assays and Transwell assays were used to examine the proliferation and invasion of HepG2 and HCCLM3 cells after the above transfection. **P < 0.01.

For determining the specific systems of miR-448 activity within HCC cells, this study carried out bioinformatics investigation for identifying the possible targets of miR-448. This study reported one miR-448 binding site inside ROCK1′s 3′-UTR (Figure 5A), as taken to carry out the following verifying process for its critical oncogenic roles in HCC. According to RT-PCR and Western blot tests, ROCK1 was overexpressed within HCC cells compared with LO2 cells (Figure 5B). According to functional tests, knockdown of ROCK1 noticeably inhibited HCC cells from being proliferated and invaded (Figures 5C–E). To verify the ROCK1 aiming based on miR-448, the present study carried out the luciferase activity test. As opposed to the control the cotransfection of miR-448 with WT-3′UTR in HepG2 and HCCLM3 cells limited luciferase activity noticeably, whereas Mut-3′UTR luciferase activity remained unchanged (Figure 5F). In addition, overexpression of miR-448 distinctly inhibited the expression of ROCK1 at both mRNA and protein levels (Figure 5G). To explore whether PITPNA-AS1 subsequently controls ROCK1 expression, we performed rescue experiments and observed that miR-448 knockdown reversed the suppression of PITPNA-AS1 knockdown on the expression of ROCK1 (Figure 6A). Moreover, a series of functional assays confirmed that miR-448 knockdown reversed the suppression of PITPNA-AS1 knockdown on the proliferation and invasion (Figures 6B–D). Taken together, PITPNA-AS1 aggravated the oncogenicity of HCC cells by regulating the miR-448/ROCK1 axis.

## Discussion

Recently, growing studies have suggested the potential of lncRNAs used as novel diagnostic and prognostic biomarkers for HCC, such as lncRNA p34822 and lncRNA SNHG1 (13, 14). These functional lncRNAs were shown to be positively associated with metastasis and relapses, which encouraged us to further identify novel biomarkers for HCC. In this study, we found that PITPNA-AS1 was distinctly overexpressed in HCC based on TCGA datasets and RT-PCR data from our cohort, which was consistent with previous findings by Sun et al. (11). We also observed that patients with high PITPNA-AS1 expression exhibited advanced clinical stages, positive metastasis and poor prognosis than those with low PITPNA-AS1 expression. Previously, the prognostic value of PITPNA-AS1 was also reported in colorectal cancer and cervical cancer (12, 15). Our study firstly provided clinical evidence that PITPNA-AS1 may be used as a novel biomarker. However, the sample size is relatively small, large clinical trials are needed to conduct.

Recently, PITPNA-AS1 was found for facilitating colorectal cancer cells' proliferation and metastasis through miRNA-129-5p/HMGB1 axis (12). In lung squamous cell carcinoma, PITPNA-AS1 was also observed to boost tumor cells' proliferating and migrating processes via recruiting TAF15 for stabilizing HMGB3 mRNA (10). Sun et al. also reported the oncogenic roles of PITPNA-AS1 within HCC (11). These findings suggested that the function of PITPNA-AS1 as a tumor promotor was a common event in different types of tumors. We also observed that knockdown of PITPNA-AS1 suppressed the proliferation, migration and invasion of HCC cells *in vitro* and *in vivo* studies. EMT, one primary system of tumor metastasizing process, results in the loss of cell-cell adhesion and promotes the ability to migrate and invade (16). Here, we confirmed the EMT progress was suppressed after the knockdown of PITPNA-AS1. Our findings, together with previous findings, suggested PITPNA-AS1 as an oncogene in HCC.

When the effect exerted by PITPNA-AS1 to be an oncogene in HCC was verified, the molecular systems influencing the changed malignant phenotypes received the exploration. It has been demonstrated that cytosolic lncRNAs are capable of modulating mRNA stable property and protein localizing process, as well as becoming microRNA sponge (17, 18). PITPNA-AS1 was suggested to have the expression in the cytoplasm and the nucleus, and PITPNA-AS1 was observed in the cytoplasm with a higher rate, suggesting PITPNA-AS1 may serve as a ceRNA. Then, PITPNA-AS1 was evidenced to be a ceRNA for miR-448 inside the cytoplasm and suppressed miR-448 expressions. Previously, several studies have demonstrated that miR-448 was lowly expressed in several tumors, including HCC, and overexpression of miR-448 was observed to promote tumor metastasis of HCC (19, 20). We also observed the oncogenic roles of miR-448 in HCC, which was consistent with previous findings. Thus, these results suggested PITPNA-AS1 may exhibit its effects via sponging miR-448.

ROCK1, a vital downstream effecting element pertaining to the small GTPase RhoA, refers to one serine/threonine kinase, mediating a range of cellular responding processes (e.g., cell proliferating process, growing process, and apoptosis based on microtubule network organizing process and influences on the cytoskeleton) (21). ROCK1 served as an oncogene, with the involvement inside different progressions (e.g., cell migrating process, metastasis, as well as invading process) (22, 23). In HCC, it has been demonstrated that ROCK1 was overexpressed in HCC specimens and promoted the proliferation and metastasis of HCC cells (24–28). However, the potential mechanisms involved in ROCK1 function remained largely unclear. In this study, we found ROCK1 may be a target of miR-448. Previously, miR-448 was reported to directly target ROCK1, thus suppressing the progression of retinoblastoma (29). However, whether a similar function was observed in HCC remained unknown. We provided evidence that miR-448 targeted miR-448 and suppressed its expression. To further explore the association among PITPNA-AS1, miR-448 and ROCK1, we performed rescue experiments and found that knockdown of miR-448 reversed the suppression of PITPNA-AS1 down-regulation on the expression of ROCK1. In addition, a series of functional assays also showed that knockdown of miR-448 could reverse the inhibition of PITPNA-AS1 down-regulation on the proliferating process and metastasis of HCC cells. Therefore, PITPNA-AS1 promotes the progression of HCC via sponging miR-448 to decrease ROCK1 expression.

## Conclusions

Our study first revealed that PITPNA-AS1 is upregulated in HCC, and its overexpression may be an unfavorable prognostic factor for patients with HCC. PITPNA-AS1 could promote HCC cell proliferation and metastasis through sponging miR-448 and releasing ROCK1. The results here elucidate HCC progression and pathogenesis, probably facilitating therapeutics and diagnostics in HCC under the direction by lncRNAs.

## Data Availability Statement

The raw data supporting the conclusions of this article will be made available by the authors, without undue reservation.

## Ethics Statement

The studies involving human participants were reviewed and approved by the First Affiliated Hospital of Zhengzhou University. The patients/participants provided their written informed consent to participate in this study. The animal study was reviewed and approved by the First Affiliated Hospital of Zhengzhou University.

## Author Contributions

Q-fW and M-bC designed the study and wrote the manuscript. Q-fW and Q-lW conducted the experiments and data analysis. Q-lW provided technical support. All authors read and approve the final manuscript.

## Conflict of Interest

The authors declare that the research was conducted in the absence of any commercial or financial relationships that could be construed as a potential conflict of interest.
